# Tumor-to-Tumor Metastasis of Epidermal Growth Factor Receptor (EGFR)-Mutated Lung Adenocarcinoma to a Meningioma 14 Years After Curative Lobectomy: A Case Report

**DOI:** 10.7759/cureus.97093

**Published:** 2025-11-17

**Authors:** Tomoaki Yoneda, Tomoyuki Araya, Takayuki Higashi, Ryo Hara, Toshiyuki Kita

**Affiliations:** 1 Respiratory Medicine, National Hospital Organization Kanazawa Medical Center, Kanazawa, JPN

**Keywords:** adenocarcinoma of the lung, epidermal growth factor receptor (egfr), late recurrence, meningioma, tumor-to-tumor metastasis

## Abstract

Tumor-to-tumor metastasis is a rare oncologic phenomenon in which one primary malignancy metastasizes into another distinct neoplasm. While meningiomas are the most common recipients, this route of recurrence has not been reported in epidermal growth factor receptor (*EGFR*)-mutated lung adenocarcinoma. A 74-year-old female patient, who had remained disease-free for 14 years after undergoing right upper lobectomy for stage IA (pT1N0M0) lung adenocarcinoma, presented with headache and vomiting. Brain MRI demonstrated a 30 × 35 mm mass in the left parietal lobe with both solid and cystic portions abutting the dura and associated with surrounding edema, which was subsequently resected. Histopathological examination showed an admixture of meningioma and adenocarcinoma components, consistent with tumor-to-tumor metastasis. Immunohistochemistry revealed adenocarcinoma cells positive for thyroid transcription factor-1, Napsin A, and cytokeratin 7, supporting a pulmonary origin. Molecular testing identified an *EGFR* exon 19 deletion in both the intracranial lesion and the primary lung adenocarcinoma resected 14 years earlier, confirming a shared clonal origin. Following diagnosis, the patient received postoperative radiation therapy followed by osimertinib, and she remains recurrence-free three years after surgery. This case highlights an exceptionally rare pattern of recurrence, tumor-to-tumor metastasis of *EGFR*-mutated lung adenocarcinoma after right upper lobectomy into a meningioma, occurring 14 years after curative surgery. Oncologists should recognize that *EGFR*-mutated lung cancer can recur after an exceptionally long disease-free interval through rare metastatic patterns such as tumor-to-tumor metastasis.

## Introduction

Tumor-to-tumor metastasis is a rare oncologic phenomenon in which one neoplasm serves as the recipient of metastatic deposits from another primary tumor [1]. Since Fried first described tumor-to-tumor metastasis originating from lung cancer in 1930 [2], over 685 cases have been reported, with the most frequent pattern involving metastasis from breast or lung cancer to a meningioma [3]. A recent systematic review and meta-analysis by Turner et al. further confirmed that meningioma is the most common recipient tumor in tumor-to-tumor metastasis, likely owing to its vascularity and unique biological microenvironment [4]. Despite meningioma being the most common recipient tumor, tumor-to-tumor metastasis remains exceptionally rare in lung cancer, and recurrence of epidermal growth factor receptor (*EGFR*)-mutated adenocarcinoma through this route has not been previously reported. In addition, the majority (72.7%) of late recurrences occurring more than five years after lung cancer surgery have been reported in *EGFR*-positive non-small-cell lung cancer (NSCLC) [5], highlighting the long-term recurrence potential of *EGFR*-mutated tumors. A comprehensive literature search was performed using PubMed and Google Scholar with the keywords “tumor-to-tumor metastasis,” “lung adenocarcinoma,” and “meningioma.” No previous reports describing tumor-to-tumor metastasis of *EGFR*-mutated lung adenocarcinoma to a meningioma were identified. Here, we describe the first case of *EGFR*-mutated lung adenocarcinoma that recurred 14 years after curative lobectomy as a tumor-to-tumor metastasis to an intracranial meningioma.

## Case presentation

A 74-year-old woman presented with headache, nausea, and vomiting. She was a lifelong nonsmoker, consumed alcohol occasionally, and had no history of allergy or regular medication use. There was no notable family history. She had worked as a homemaker and had no known occupational exposure to dust, chemicals, or radiation. She had undergone a right upper lobectomy 14 years earlier for stage IA (pT1N0M0) lung adenocarcinoma. At the time of surgery, a brain CT performed at another hospital revealed no intracranial abnormalities, as documented in the medical record. The original images were not available for review. She had remained disease-free on routine follow-up.

On admission, physical examination was unremarkable, with normal vital signs and no neurological deficits; her Karnofsky Performance Status (KPS) was 70 at that time. Laboratory tests showed almost normal blood counts and biochemistry results, except for slight lymphopenia, an elevated neutrophil ratio, and mildly increased tumor markers, including carcinoembryonic antigen (6.8 ng/mL) and cytokeratin 19 fragment (4.1 ng/mL).

**Figure 1:**
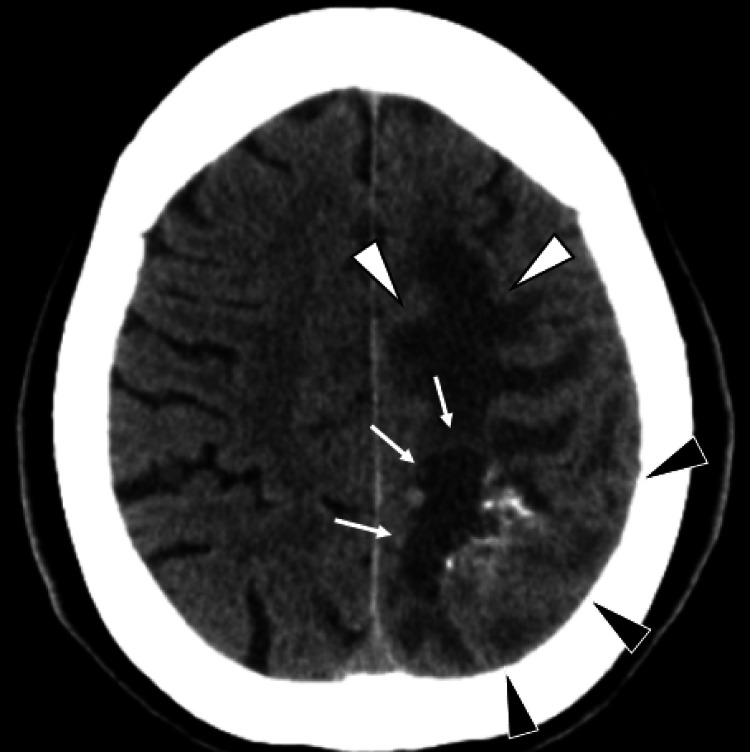
Brain CT findings. Axial non-contrast-enhanced brain CT image showing a 30 × 35 mm tumor in the left parietal lobe composed of an isodense solid component (black arrowheads) and a hypodense cystic component (white arrows). Peritumoral edema is visible (white arrowheads), but no midline shift is present.

Non-contrast-enhanced brain CT demonstrated a 30 × 35 mm mass in the left parietal lobe composed of an isodense solid component and a hypodense cystic component, accompanied by marked peritumoral edema without midline shift (Figure 1).

**Figure 2:**
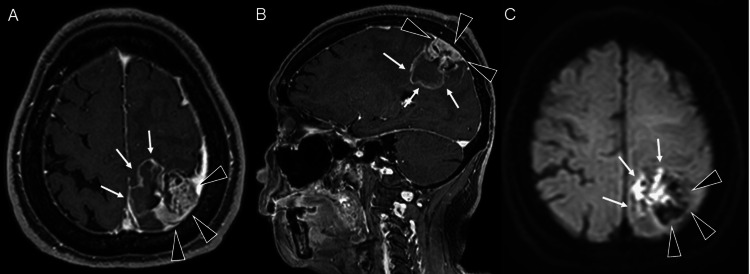
Brain MRI findings. Gadolinium-enhanced T1-weighted images showing a left parietal lobe tumor broadly attached to the dura with associated dural thickening. The dural-based solid component (A, axial; B, sagittal; black arrowheads) exhibits heterogeneous enhancement, whereas the deeper portion shows irregular morphology with marginal and poor internal enhancement, suggestive of necrosis (white arrows). On diffusion-weighted imaging (C), the dural-based solid component demonstrates low signal intensity (black arrowheads), while the deeper portion appears hyperintense (white arrows).

The lesion showed broad dural attachment with associated dural thickening. On MRI, gadolinium-enhanced T1-weighted images showed that the dural-based solid portion exhibited heterogeneous enhancement, whereas the deeper component showed irregular morphology with rim-predominant and poor internal enhancement, suggestive of necrosis (Figures 2A, 2B). On diffusion-weighted imaging, the dural-based solid portion appeared hypointense, while the deeper component was hyperintense (Figure 2C).

No other metastatic sites were detected on chest-to-pelvis CT. 18F-fluorodeoxyglucose positron emission tomography/computed tomography demonstrated intense uptake in the left parietal lesion, with a maximum standardized uptake value of 11.9. Based on these imaging findings, an atypical meningioma or solitary fibrous tumor was suspected. Therefore, craniotomy and complete resection of the brain lesion were performed for definitive diagnosis and treatment, at which time her KPS was 70.

**Figure 3:**
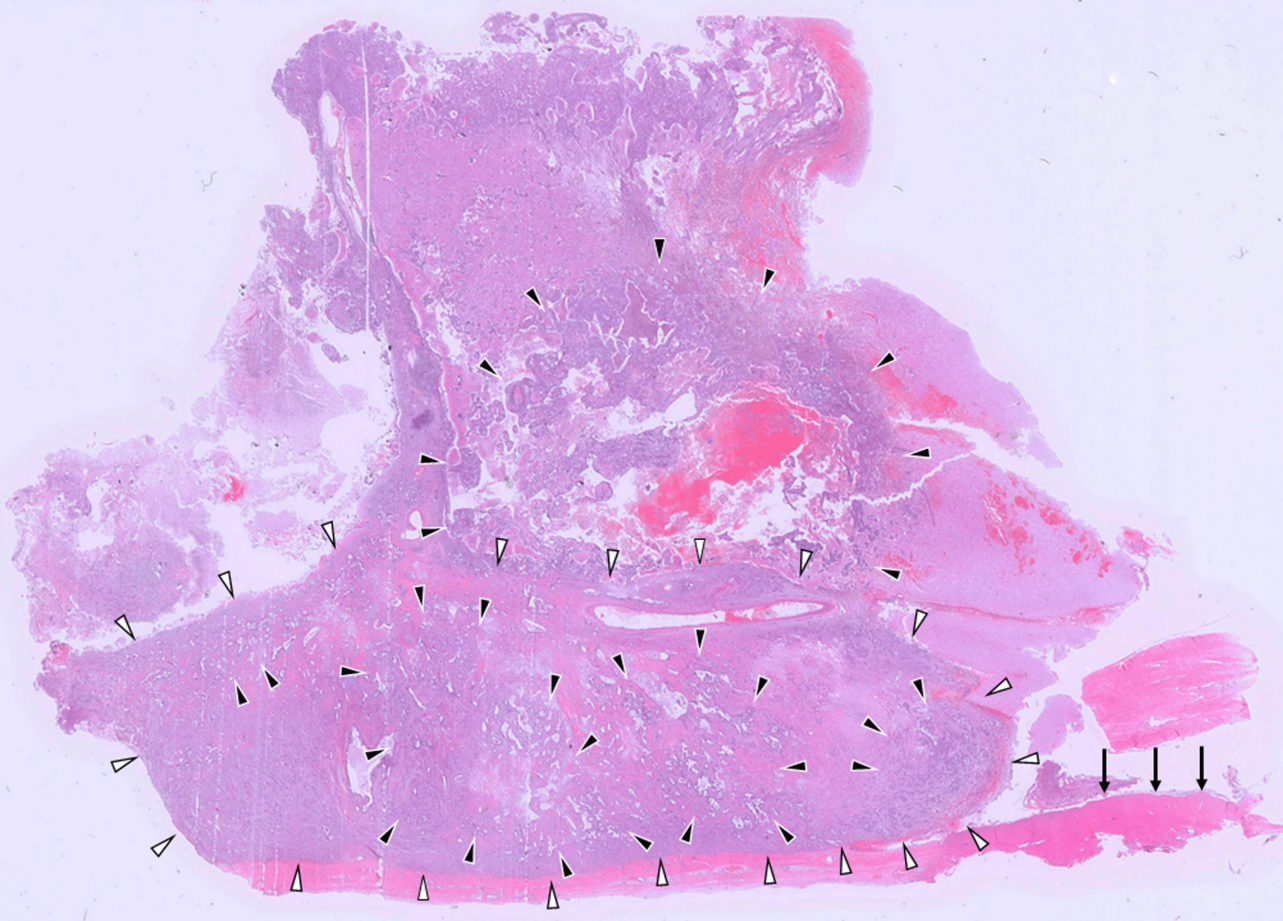
Loupe view of the resected brain tumor in the left parietal lobe stained with hematoxylin and eosin (H&E). Histopathological examination of the resected brain tumor shows a fibrous meningioma (white arrowheads) attached to the dura mater (black arrows). Adjacent to the meningioma, on the parenchymal side, adenocarcinoma with marked necrosis and hemorrhage (black arrowheads) is observed. The adenocarcinoma has extensively infiltrated into the meningioma, spreading widely within it and creating a mingled appearance consistent with tumor-to-tumor metastasis.

Histopathological examination revealed two distinct but admixed components: a fibrous meningioma and a moderately differentiated adenocarcinoma extensively infiltrating within the meningioma tissue (Figure 3).

**Figure 4:**
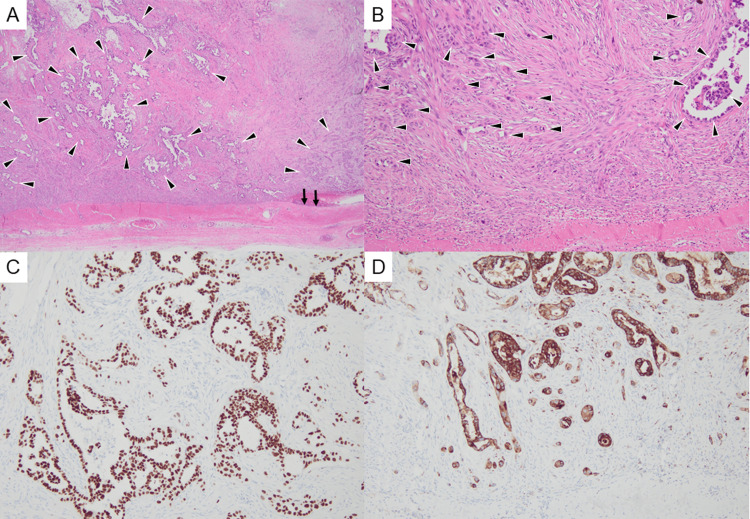
Histopathological and immunohistochemical findings of the resected brain tumor. (A) Hematoxylin and eosin (H&E) staining at ×20 magnification shows a fibrous meningioma adjacent to the dura mater (black arrows). The meningioma is composed of elongated spindle cells with sparse cytoplasm arranged in fascicular and whorled patterns, accompanied by collagenous stroma and small blood vessels. Within the meningioma, moderately to poorly differentiated adenocarcinoma cells (black arrowheads) infiltrate extensively, forming irregular tubular, papillary, solid, and alveolar structures that intermix with the meningioma tissue. This finding represents tumor-to-tumor metastasis, in which adenocarcinoma has metastasized into the meningioma. (B) H&E staining at ×100 magnification demonstrates similar infiltration of adenocarcinoma cells (black arrowheads) widely spreading within the meningioma. (C) Immunohistochemical staining for thyroid transcription factor-1 shows nuclear positivity in adenocarcinoma cells, whereas the surrounding meningioma cells are completely negative. (D) Immunohistochemical staining for Napsin A (×100) also shows cytoplasmic positivity in adenocarcinoma cells, while the background meningioma cells remain entirely negative.

The meningioma component consisted of elongated spindle cells with sparse cytoplasm arranged in fascicular and whorled patterns and exhibited low mitotic activity, consistent with a fibrous meningioma. In contrast, the adenocarcinoma component, composed of moderately to poorly differentiated cells, displayed irregular tubular, papillary, solid, and alveolar structures infiltrating widely into the meningioma (Figures 4A, 4B). Immunohistochemical staining demonstrated that the adenocarcinoma cells were positive for thyroid transcription factor-1, Napsin A, cytokeratin 7, and epithelial membrane antigen (EMA), and negative for cytokeratin 20, findings consistent with pulmonary origin (Figures 4C, 4D).

The meningioma component, on the other hand, was positive for progesterone receptor and weakly positive for EMA, confirming its benign nature.

Molecular analysis of the resected brain tumor using the AmoyDx mutation detection panel [6] revealed an exon 19 deletion. Subsequently, the archived primary lung specimen from 14 years earlier was retrieved and analyzed using the Cobas® EGFR Mutation Test v2 [7], which confirmed the presence of the same exon 19 deletion. These results established that the intracranial lesion represented a late recurrence of the patient’s original *EGFR*-mutated lung adenocarcinoma. Based on the imaging findings together with these molecular analyses, the final diagnosis was tumor-to-tumor metastasis of lung adenocarcinoma to a meningioma.

Two months after surgery, when her KPS had improved to 90, intensity-modulated radiotherapy (45 Gy in 15 fractions) was initiated to the resection site, followed by oral osimertinib (80 mg/day). The patient remains alive and recurrence-free 35 months after surgery.

## Discussion

The present case demonstrates an exceptionally rare pattern of recurrence in lung cancer, tumor-to-tumor metastasis occurring 14 years after curative resection. Such an extraordinarily long disease-free interval is highly unusual, as most tumor-to-tumor metastases develop within two years of the primary diagnosis [3]. Several large cohort studies have suggested that NSCLC harboring *EGFR* mutations is prone to late recurrence even after complete resection [5,8-10]. In *EGFR*-mutated NSCLC, the risk of recurrence persists beyond five years postoperatively, and late recurrences are more frequent than in *EGFR* wild-type cases. The recurrence risk has been reported to extend up to eight to nine years after surgery, with most late events occurring in patients with EGFR-mutated tumors. Moreover, EGFR-mutated cases tend to relapse with distant metastases, particularly to the brain and bone. Taken together with these previous findings, this case indicates that *EGFR*-mutated lung adenocarcinoma may maintain a long-term potential for reactivation even after an extended period of clinical remission, leading to recurrence through uncommon metastatic routes such as tumor-to-tumor metastasis to a meningioma.

Meningioma has been reported as the most common recipient tumor in cases of tumor-to-tumor metastasis [3], and several biological factors are thought to contribute to this susceptibility, including rich vascularization, low metabolic activity, immunological tolerance, expression of adhesion molecules such as E-cadherin and mesothelin, high collagen and lipid content, and the presence of hormone receptors (estrogen and progesterone) [11-14].

Although the possibility of a collision tumor was considered based on imaging findings, histopathological examination clearly demonstrated that adenocarcinoma cells had extensively and deeply infiltrated into the meningioma tissue, forming a nearly admixed interface between the two components. This feature distinguishes our case from a collision tumor and provides strong evidence supporting true tumor-to-tumor metastasis, in contrast to previously reported collision tumor cases involving meningioma and pituitary adenoma [15].

Given the growing number of long-term survivors of *EGFR*-mutated lung cancer due to advances in targeted therapy, clinicians should be aware that very late recurrences can occur through atypical metastatic routes such as tumor-to-tumor metastasis. Maintaining clinical vigilance and considering this rare entity in the differential diagnosis of intracranial tumors are crucial for accurate diagnosis and optimal management, particularly in cases of very late postoperative recurrence.

## Conclusions

We report the first case of tumor-to-tumor metastasis from *EGFR*-mutated lung adenocarcinoma to a meningioma occurring 14 years after curative lobectomy. Molecular analysis was key in confirming the metastatic relationship. Awareness of this rare recurrence pattern is essential, as prolonged survival of patients with *EGFR*-mutated lung cancer may increase the likelihood of encountering such atypical metastatic phenomena.
